# Circadian variation of post-operative complications in lung resection surgery

**DOI:** 10.3389/fphys.2026.1818583

**Published:** 2026-05-20

**Authors:** Marek Lukes, Kristian Brat, Jan Hruda, Zdeněk Chovanec, Michal Svoboda, Lyle Olson, Ivan Cundrle

**Affiliations:** 1 Faculty of Medicine, Masaryk University, Brno, Czechia; 2 Centre of Cardiovascular and Transplant Surgery, Brno, Czechia; 3 Center for Pulmonology and Interventional Bronchology, Masaryk Memorial Cancer Institute, Brno, Czechia; 4 Department of Anesthesiology and Intensive Care, St. Anne’s University Hospital Brno, Brno, Czechia; 5 First Department of Surgery, St. Anne’s University Hospital, Brno, Czechia; 6 Institute of Biostatistics and Analyses, Ltd., Brno, Czechia; 7 Department of Cardiovascular Diseases, Mayo Clinic, Rochester, MN, United States

**Keywords:** circadial rhythm disorders, lung resection, postoperative cardiovascular complications, postoperative pulmonary complications (PPC), spiroergometry

## Abstract

**Introduction:**

Studies have revealed circadian rhythms affect many organ systems and time of injury can impact healing, lung function, and inflammation. We hypothesized operative time may impact post-operative risk. Accordingly, the aim of this study was to compare post-operative complications in patients whose surgery started and finished before 12pm (Group 1); after 12pm (Group 2); and started before 12pm and finished after 12pm (Group 3).

**Methods:**

Patients from two previous prospective studies were included in this *post-hoc* analysis. Inclusion criteria were indication for lung resection surgery (confirmed or highly suspicious tumor), age ≥18 and ability to undergo cardiopulmonary exercise testing (CPET). Exclusion criterion was inability to perform surgery (either low predicted postoperative oxygen consumption or inoperability). All patients underwent pre-operative CPET and spirometry. Post-operative pulmonary (PPC) and cardiovascular complications (PCC) were prospectively assessed from the hospital stay up to 30 days.

**Results:**

Data from 497 patients were analyzed. On comparison of groups, there were differences in BMI (highest in Group 3), surgery duration (lowest in Group 1) and frequency of lobectomies (lowest in Group 1). Incidence of PPC was lowest in Group 1 (n=17; 9%) compared to n=17 (21%) in Group 2 and n=37 (17%) in Group 3 (p=0.01). Incidence of PCC was lowest in Group 1 n=25 (13%) compared to n=19 (23%) in Group 2 and n=46 (21%) in Group 3 (p=0.05). Multivariate logistic regression adjusted for confounders (BMI, surgery duration and lobectomy) showed time of surgery after 12pm was independently associated with PPC with OR = 2.17 (95% CI 1.07-4.41); p=0.03.

**Conclusion:**

Time of surgery may impact the incidence of PPC in lung resection surgery patients with highest risk in patients undergoing the surgery after 12pm.

## 1 Introduction

Many biological processes demonstrate circadian rhythm which regulates wakefulness and sleep, as well as food intake and energy expenditure, endocrine signaling, and autonomic nervous system and immune responses (Allada and Bass, 2021; Meyer et al., 2022). Evidence suggests circadian rhythmicity plays a pivotal role in critical illness. For example, timing of noxious stimuli, including sepsis (Truong et al., 2016), burn trauma (Hoyle et al., 2017) or myocardial infarction (Eckle et al., 2024) may affect the magnitude of inflammatory response and recovery. In recent years, chronobiology has also gained increased attention in perioperative medicine as it has been shown both surgical insult and general anesthesia contribute to dysregulation of circadian rhythms (Touitou et al., 2016; van Zuylen et al., 2022). Moreover, the physiological response to surgical trauma varies throughout the day, including the degree of inflammatory activation and tissue repair capacity (Martin et al., 2018).

Timing of therapeutic interventions including major surgery may have a direct impact on post-operative recovery and outcomes. Several studies have focused on the effect of the time of elective surgery on morbidity and mortality and yielded conflicting data and inconclusive results. Elective aortic valve replacement under cardiopulmonary bypass has been associated with lower incidence of major cardiovascular events when performed in the afternoon compared to morning hours (Montaigne et al., 2018). Conversely, a recent meta-analysis of 19 studies demonstrated an increased risk of mortality among patients undergoing elective non-cardiac surgery in the evening or at night (Meewisse et al., 2024). However, the number of well-designed studies has been limited and quality of available evidence remains low.

We hypothesized that the time of day of surgery is related to post-operative complications in patients undergoing elective lung resection surgery. We conducted *post-hoc* analysis of our two previous prospective studies. The aim of this study was to compare pulmonary and cardiovascular complications in three groups of patients who underwent surgery in the morning, in the afternoon, and those whose operation began before noon and ended in the afternoon.

## 2 Methods

### 2.1 Patients

This is a *post-hoc* analysis of two prospective, multicenter studies conducted at medical institutions in the Czech Republic (St. Anne’s University Hospital in Brno and University Hospital Brno). The study population consisted of adult patients undergoing lung resection due to either confirmed or highly suspected lung cancer, who were able to perform cardiopulmonary exercise testing (CPET). Patients were excluded if they had inoperable tumors and/or were deemed unfit for surgery due to a low predicted postoperative peak VO_2_ (< 35% or < 10 ml/kg/min) (Brunelli et al., 2009). Both studies were registered at ClinicalTrials.gov (NCT03498352 and NCT04826575) and were conducted in accordance with the Declaration of Helsinki, following approval by the Ethics Committee of St. Anne’s University Hospital (Ref. No.: 19JS/2017, 2G/2018, 03G/2021) and the Ethics Committee of University Hospital Brno (Ref. No.: 150617/EK, 14-100620/EK). Written informed consent was obtained from all participants.

Patients were categorized according to the relation between the time of surgery and noon (12pm) into three groups: Group 1, surgery starting before 12pm and ending before 12pm; Group 2, surgery starting after 12pm; and Group 3, surgery starting before 12pm and ending after 12pm. The 12 pm cutoff was chosen for pragmatic reasons.

### 2.2 Pulmonary function tests

All patients underwent pulmonary function testing (PFT) in accordance with the guidelines set by the European Respiratory Society (ERS) and the American Thoracic Society (ATS) (Miller et al., 2005). Spirometric assessments were carried out using the ZAN100 system (nSpire Health Inc., Longmont, USA), while diffusing capacity for carbon monoxide (DL_CO_) was measured with the PowerCube Diffusion+ device (Ganshorn Medizin Electronic GmbH, Niederlauer, Germany). Spirometry parameters included forced expiratory volume in one second (FEV_1_) and forced vital capacity (FVC), with results expressed as a percentage of predicted values.

### 2.3 Cardiopulmonary exercise testing

Before surgery, all participants underwent a symptom-limited cardiopulmonary exercise test (CPET) as previously described (Brat et al., 2023). Testing was performed using an electronically braked cycle ergometer (Ergoline, Ergometrics 800, Bitz, Germany) in combination with a 12-lead electrocardiograph system (AT-104, Schiller AG, Baar, Switzerland). Exhaled gases and ventilatory volumes were measured with the PowerCube-Ergo CPET system (Ganshorn Medizin Electronic GmbH, Niederlauer, Germany). The following peak exercise parameters were analyzed: oxygen consumption (VO_2_), carbon dioxide production (VCO_2_), tidal volume (V_T_), breathing frequency (f_b_), minute ventilation (V_E_), ventilatory efficiency (V_E_/VCO_2_ slope), and the dead space to tidal volume ratio (V_D_/V_T_).

### 2.4 Pulmonary postoperative complications

Pulmonary complications were defined similarly as in previous thoracic surgery studies (Stéphan et al., 2000; Licker et al., 2006; Torchio et al., 2010; Brunelli et al., 2012; Brat et al., 2023). These complications included pneumonia (diagnosed based on chest X-ray findings of infiltrates accompanied by clinical signs such as fever, leukocytosis or leukopenia, and purulent sputum), atelectasis (identified on chest X-ray and confirmed by bronchoscopy), respiratory failure (necessitating either non-invasive or invasive mechanical ventilation), and acute respiratory distress syndrome (ARDS). Complications were prospectively monitored during the 30-day postoperative hospital period.

### 2.5 Postoperative cardiovascular complications

Cardiovascular complications were defined according to prior studies (Bennett-Guerrero et al., 1999; Mazur et al., 2022). These complications included new onset arrhythmias (atrial fibrillation or supraventricular tachycardia persisting for at least 10 seconds (Camm et al., 2010)), pulmonary edema (diagnosed based on chest X-ray findings and clinical presentation), hypotension (evidenced by the use of catecholamines and/or administration of intravenous fluids exceeding 200 mL/h), myocardial infarction or injury (confirmed by coronary angiography and/or new electrocardiographic changes in conjunction with elevated blood troponin levels), heart failure (increased serum brain natriuretic peptide concentrations and/or the use of inotropic agents (McDonagh et al., 2021)), pulmonary embolism (verified through CT angiography or strongly suggestive signs of right heart dysfunction observed via echocardiography), stroke (defined by a new neurological deficit and CT confirmation of cerebral infarction—either ischemic or hemorrhagic in nature (Mashour et al., 2014)), and cardiopulmonary resuscitation. Additionally, the length of stay in the intensive care unit (ICU) and the total hospital stay were recorded.

### 2.6 Statistics

Normality of data distribution was assessed using the Shapiro–Wilk test. Differences between groups were evaluated using one-way analysis of variance (ANOVA) or the Kruskal–Wallis rank-sum test, followed by either the Tukey HSD *post-hoc* test or the Mann–Whitney U test, as appropriate. Categorical variables were compared using the chi-square (χ²) test. To identify independent predictors of postoperative pulmonary and cardiovascular complications, multivariate logistic regression analysis was performed. Only variables showing statistically significant differences between groups and without strong intercorrelations were included in the model. Group 3 was selected as the reference category because it represented the largest, most representative, and clinically meaningful mixed-exposure category subgroup in the cohort, thereby providing the most stable comparator for adjusted estimates in the presence of unequal group sizes (Frøslie et al., 2010; Hansen and Lian, 2016). Data are presented as mean ± standard deviation (SD) or median with interquartile range (IQR), depending on distribution. A p-value < 0.05 was considered statistically significant. All statistical analyses were performed using Statistica software version 14.0 (StatSoft Inc., Prague, Czech Republic).

## 3 Results

Four hundred ninety-seven patients were included in this *post-hoc* analysis. Basic group comparisons are shown in **Table 1**. Between groups, there were significant differences in BMI, duration of surgery, frequency of lobectomies, pneumonectomies and wedge resections (**Table 1**). PFTs and CPET comparisons are shown in **Table 2**. There were no significant differences between all 3 groups.

**Table 1:** Subject characteristics by group.

Parameter	Group 1 (n=194)	Group 2 (n=81)	Group 3 (n=222)	p ANOVA/χ2
Male No (%)	103 (53)	48 (59)	126 (57)	0.59
Age (years)	66 (57-71)	66 (58-72)	67 (59-72)	0.33
BMI (kg/m^2^)	27 (23-30)	28 (24-31)	28 (25-33)**	0.01
S-MPM	6 (4-6)	6 (4-6)	6 (4-6)	0.34
COPD No (%)	49 (25)	24 (30)	55 (25)	0.68
Surgery				
Surgery duration (min)	100 (65-150)	130 (100-170)**	190 (135-245)**§§	<0.01
Thoracotomy No (%)	87 (45)	42 (52)	116 (52)	0.28
Lobectomy No (%)	65 (34)	39 (48)*	137 (62)**§	<0.01
Bilobectomy No (%)	3 (2)	2 (2)	12 (5)	0.08
Pneumonectomy No (%)	1 (1)	0	9 (4)*	0.01
Wedge resection No (%)	125 (64)	41 (51)*	64 (29)**§§	<0.01

BMI, body mass index; COPD, Chronic obstructive pulmonary disease; S-MPM, Surgical Mortality Probability Model; *, p<0.05 vs group 1; **, p<0.01 vs group 2; §, p<0.05 vs group 1; §§, p<0.01 vs group 2.

**Table 2:** Pulmonary function tests and cardiopulmonary exercise testing comparison.

Parameter	Group 1 (n=194)	Group 2 (n=81)	Group 3 (n=222)	p ANOVA
Pulmonary function tests				
FEV_1_ (%)	93 ± 20	91 ± 18	91 ± 19	0.81
FVC (%)	96 (83-106)	96 (88-106)	93 (83-106)	0.65
FEV_1_/IVC (%)	79 (73-84)	78 (71-83)	78 (71-83)	0.15
DL_CO_ (%)	86 ± 23	82 ± 20	84 ± 24	0.52
Peak exercise ventilation and gas exchange				
VO_2_ (ml/kg/min)	18.7 (15.8-23)	19.1 (15.9-22.5)	18.9 (15.7-23.0)	0.99
VCO_2_ (l/min)	1.60 (1.29-2.01)	1.68 (1.38-2.00)	1.62 (1.31-2.00)	0.62
V_E_ (l/min)	53 (44-65)	57 (45-68)	55 (44-69)	0.47
V_T_ (l)	1.68 (1.34-2.01)	1.75 (1.43-2.10)	1.74 (1.35-2.14)	0.42
f_b_	33 (29-37)	33 (29-36)	33 (29-37)	0.72
V_D_/V_T_	0.22 (0.17-0.26)	0.23 (0.16-0.26)	0.21 (0.17-0.27)	0.98
V_E_/VCO_2_ slope	30 (26-33)	29 (25-34)	30 (26-35)	0.11

DL_CO_, diffusing lung capacity for carbon monoxide; FEV_1_, forced expiratory volume in one second; FVC, forced vital capacity; f_b_, breathing frequency; V_D_/V_T_, dead space to tidal volume ratio; V_E_, minute ventilation; V_E_/VCO_2_ slope, ventilatory efficiency slope; VCO_2_, carbon dioxide output; VO_2_, oxygen consumption; V_T_, tidal volume.

Post-operative pulmonary and cardiovascular complications and outcomes are shown in **Table 3**. Compared to Group 1, a composite of pulmonary and cardiovascular complications was significantly higher in both Group 2 and 3. For pulmonary complications, bronchopneumonia was significantly more frequent in Group 2 and 3, whereas respiratory failure, atelectasis and ARDS were more frequent in Group 2 compared to group 1. For cardiovascular complications, arrythmias were more frequent in Group 2, compared to both group 1 and 3. Hospital and ICU length of stay (LOS) were significantly longer in Group 2 and 3 compared to group 1. ICU LOS was significantly longer in group 3 compared to Group 2.

**Table 3:** Pulmonary and Cardiovascular complications type and frequency.

Parameter	Group 1 (n=194)	Group 2 (n=81)	Group 3 (n=222)	p χ2
Pulmonary complications				
Pulmonary composite	17 (9)	17 (21)**	37 (17)*	0.01
Bronchopneumonia No. (%)	14 (7)	14 (17)*	31 (14)*	0.03
Respiratory failure No. (%)	4 (2)	6 (7)*	4 (2)§	0.02
Atelectasis No. (%)	2 (1)	6 (7)**	9 (4)	0.02
ARDS No. (%)	0	2 (2)*	1 (0.5)	0.05
Cardiovascular complications				
Cardiovascular composite	25 (13)	19 (23)*	46 (21)*	0.05
Hypotension No (%)	14 (7)	11 (14)	28 (13)	0.13
Heart failure No (%)	0	1 (1)	1 (0.5)	0.05
Lung edema No (%)	1 (1)	1 (1)	0	0.31
Acute myocardial infarction No (%)	0	1 (1)	0	0.08
Arrhythmia No (%)	14 (7)	17 (21)**	24 (11)§	<0.01
Pulmonary embolism No (%)	0	1 (1)	3 (1)	0.27
Cardiopulmonary resuscitation No (%)	1 (1)	2 (2)	1 (0.5)	0.19
Ischemic stroke No (%)	1 (1)	1 (1)	0	0.31
Outcome				
Hospital LOS (days)	6 (5-8)	7 (5-9)**	7 (6-10)**	<0.01
ICU LOS (days)	3 (2-4)	3 (2-5)*	4 (2-5)**§	<0.01
ICU readmission No (%)	5 (3)	4 (5)	6 (3)	0.54
30 days mortality No (%)	3 (2)	3 (4)	3 (1)	0.37

ICU, intensive care unit; LOS, length of stay; *p<0.05 vs group 1; **p<0.01 vs group 2; §p<0.05 vs group 1; §§p<0.01 vs group 2.

Logistic regression models for PPC and PCC are shown in **Table 4**. BMI, duration of surgery and lobectomy were significantly associated with the development of PPC. Regarding the time of start of surgery, only start after 12pm was significantly associated with the development of PPC. For PCC, only the duration of surgery was significantly associated with the development of PCC. Time of surgery was not associated with the development of PCC.

**Table 4:** Multivariate logistic regression models - PPC a PCC.

Parameter	PPC		PCC	
	OR (95% CI)	p	OR (95% CI)	p
BMI	1.06 (1.01-1.12)	0.01	0.98 (0.93-1.02)	0.33
Duration of surgery	1.01 (1.00-1.01)	<0.01	1.00 (1.00-1.01)	<0.01
Lobectomy	2.31 (1.28-4.19)	0.01	1.64 (0.97-2.75)	0.06
before 12pm	1.10 (0.53-2.26)	0.80	1.25 (0.66-2.39)	0.49
after 12pm	2.17 (1.07-4.41)	0.03	1.90 (0.97-3.72)	0.06

BMI, body mass index; PCC, post-operative cardiovascular complications; PPC, post-operative pulmonary complications.

## 4 Discussion

The major finding of this study was that incidence of both PPC and PCC were higher in elective thoracic surgery patients whose surgery started or ended after 12pm. In contrast, patients whose surgeries started in the morning and ended before 12pm had the lowest incidence of PPC and PCC. Multivariate logistic regression demonstrated lobectomy, surgery duration and time of surgery (specifically start of surgery after 12pm) were independently associated with development of PPC. In contrast, PCC did not show an independent association with time of surgery.

The incidence of PPC and PCC was comparable to previous studies. In our cohort, the overall incidence of postoperative pneumonia across all 3 groups of patients was 11.9%, incidence of ARDS 0.6% and incidence of arrhythmia 11.1%. This corresponds to previous studies showing the incidence of pneumonia 10.7% (Giudici et al., 2025), ARDS rates up to 0.7% (Piccioni et al., 2023) and incidence of NOAF 11.8% (Ivanovic et al., 2014).

In this study, PPCs (including pneumonia, respiratory failure, atelectasis and ARDS) were independently associated with start of surgery after 12pm. In previous studies, in non-thoracic surgery patients, no correlation between time of surgery and PPC incidence was found (Lu et al., 2017; Ishiyama et al., 2019). An explanation might be the incidence of PPC, which is highest in thoracic surgery patients (C et al., 2025), enabling detection.

Prior animal studies have shown association of lung functions and circadian rhythm. Time of day has been shown to significantly impact the level of inflammatory response induced by mechanical ventilation (Felten et al., 2023). Lung injury, defined by hypoxemia or pulmonary permeability, was more severe when mice were ventilated during their resting phase compared to animals ventilated at the beginning of their active phase (Felten et al., 2023). In our studies, we observed higher incidence of ARDS in afternoon surgeries. The release of inflammatory cytokines as well as recruitment and activation of innate immune cells also demonstrates circadian rhythm (Coiffard et al., 2019). Indeed, immune response has been shown to be strongest in the morning (Zhang et al., 2021) and may account for higher incidence of pneumonia in afternoon surgeries. Furthermore, early mobilization has been shown to reduce atelectasis incidence after surgery (Moradian et al., 2017). Higher incidence of atelectasis observed in the afternoon surgery may be explained by postponed rehabilitation until the next day.

Post-operative arrhythmia was more frequent in the afternoon (Group 2). Although the incidence of acute myocardial infarction or severe arrhythmias is generally more frequent in the morning (as a result of increased autonomic nervous system activity, endogenous catecholamines concentration or platelet aggregability (Muller et al., 1989)), supraventricular arrhythmias are more frequent in the afternoon (Black et al., 2019) which is in agreement with our observation.

We were not able to prove any independent association between the time of the day and frequency of cardiovascular events. These results are in contrast with previously published studies. Montaigne et al. observed better tolerance to ischemia/reperfusion and lower risk of perioperative myocardial injury (PMI) in on-pump aortic valve replacement (AVR) performed in the afternoon as compared with patients undergoing the same procedure in the morning (Montaigne et al., 2018). However, this observation was not confirmed in subsequent non-randomized and retrospective studies (Baik et al., 2019; Götte et al., 2020). A recent meta-analysis of 5 studies and more than 18–000 patients undergoing uncomplicated isolated coronary artery bypass grafting or isolated AVR did not find any evidence that daytime affects PMI or mortality after cardiac surgery (Fudulu et al., 2021). Similarly, a cohort study conducted to assess daytime variation in PMI following non-cardiac surgery did not demonstrate an association between the time of surgery and adverse cardiac events (du Fay de Lavallaz et al., 2019).

The observed association between PPC and the timing of surgery may also be explained by healthcare worker and system related factors (Meewisse et al., 2024). Surgeon fatigue, reduced resource availability due to lower staffing levels in the afternoon, and decreased access to supportive services such as rehabilitation later in the day are all nonnegligible factors that may have contributed to our findings. In addition, patient-related factors, including ASA physical status and comorbidities, may also have played a role (Meewisse et al., 2024). However, we did not observe any difference in the Surgical Mortality Probability Model (S-MPM) between the groups.

Our study has several limitations. First, as this was an observational study, selection bias may play an important role, especially regarding time of surgery. Second, although patients were recruited in two centers, both were in Czech Republic, in the south Moravian region. Therefore, generalizability to different health systems is limited. Third, we intentionally divided the cohort into three groups, as procedures crossing noon may differ from surgeries performed exclusively in the morning or exclusively in the afternoon. This approach allowed us to describe postoperative complications in a clinically relevant subgroup that was also frequently encountered in our cohort. At the same time, we acknowledge that this category partly captures longer procedures, which was indeed the case in our study. However, duration of surgery was included in the logistic regression models, and PPC remained significantly associated with the timing of surgery even after adjustment for surgical duration. Fourth, BMI differed significantly between the groups and was therefore included in the logistic regression models. Nevertheless, PPC remained significantly associated with the timing of surgery even after adjustment for BMI.

In conclusion, this study found that elective lung resection surgeries starting or ending after 12pm were associated with a higher incidence of PPC and PCC, with morning surgeries showing the lowest risk. Importantly, surgery timing (specifically starting after 12pm) was independently linked to increased PPC risk, while PCC was not independently associated with time of surgery. As this was a *post-hoc* analysis, clinical implications are hypothetical. Firstly, the findings imply that the timing of surgical procedures may influence their safety, indicating that prioritizing morning surgeries for high-risk patients or scheduling procedures based on patients’ biological rhythms could be advantageous. The study endorses further investigation within the domain of chronomedicine, especially concerning the influence of circadian rhythms on postoperative complications.

## Data Availability

The raw data supporting the conclusions of this article will be made available by the authors, without undue reservation.
